# Wisdom affinity in the general population

**DOI:** 10.1186/s40359-024-01905-4

**Published:** 2024-07-22

**Authors:** Beate Muschalla

**Affiliations:** Institute of Psychology, Technische Universität Braunschweig, Humboldtstr. 33, 38106 Braunschweig, Germany

**Keywords:** Wisdom, Life events, Stress, Coping, Representative, Capacities, Age, Gender, Mental disorder, Chronic illness

## Abstract

**Background:**

Wisdom is an important coping resource for difficult and ambiguous life situations. Wisdom trainings have been developed in clinical and non-clinical settings. What has been missing so far are representative data on wisdom affinity from the general population. These are important regarding needs assessments and identification of risk groups with low wisdom affinity and potential problems in coping with difficult and ambiguous life situations.

**Method:**

The study examined a population-representative sample of 2509 persons. Socio-demographic data, presence of chronic and mental illnesses was assessed, and wisdom attitudes by the 12-WD Wisdom Scale. The surveys were carried out by means of interviews and self-report questionnaires at the respondents’ homes, done by an experienced social research company (USUMA GmbH).

**Results:**

Only 6% of the whole sample appeared to be highly wisdom-affirmative (12-WD mean score 10 on scale 0–10), whereas 4% may appear low wisdom-affirm, due to very low agreement (12 WD mean score 0–4). Most of the moderately wisdom-affirm people had a religious denomination (70.9%), whereas only 57–59% of the high or low wisdom-affirm persons reported religious affiliations. Low wisdom-affirm were most often chronically ill (25%), with mental or physical illness in similar frequency, and had significantly more unemployment times than persons with higher wisdom scores. Wisdom affinity was independent from age, gender and age, household situation, and higher school education.

**Conclusion:**

It must be assumed that people with socio-medical risk factors also have impairments in their wisdom-related problem-solving strategies, and that these can be of interest for transdiagnostic wisdom trainings in prevention or rehabilitation, which has shown positive effects.

## Introduction

Wisdom is the capacity to cope with difficult, complex, and ambiguous life situations [1–4]. Wisdom can help to overcome negative life events. Wisdom is inherent in every human being to a greater or lesser degree. As a capacity wisdom is not a disorder-specific symptom, but a transdiagnostic quality. Like other capacities (e.g. assertiveness, or endurance), wisdom can be trained [5]. As it is not a disorder-specific phenomenon, wisdom can be diagnostically and therapeutically useful in many public health settings. Diagnosis and training can also be carried out by non-licensed professionals such as social therapists or occupational therapists, which is important for further application in prevention, treatment, and rehabilitation, may it be in clinics or outpatient settings.

Until now, wisdom has been studied as a coping resource in clinical populations. However, no systematic population-representative data on the distribution of wisdom affinity in the general population are available. Population-representative data are useful in terms of needs assessments: They allow to determine how many people are potentially in need of support, e.g. in form of wisdom education or training, in order to be better prepared for coping with unavoidably upcoming life difficulties. Representative data also make an important contribution to basic wisdom research by generating norm data that can be used for comparisons of specific (clinical) samples with the general population and, where appropriate, specific groups (e.g. age groups, employed, unemployed, age groups).

### Wisdom

Until now, there has been scientific research on wisdom for three decades [6]. Wisdom has been defined [1, 4] as expertise in dealing with difficult questions of life, such as questions of life planning, life design and life interpretation. Wisdom can be understood as a resource for coping with conflict or stress. In short, wisdom can be understood as “the capacity to solve unsolvable problems” [7]. This capacity is needed by all people every day, but especially in difficult life situations involving losses, prolonged uncertainty, economic difficulties, as well as job problems or loss, sudden unexpected or externally forced changes in one´s life situation. Many of these are problems that can hardly be influenced and are therefore practically unsolvable, such as whether one takes more care of the children and their school activities at home, or invests more time in one’s own work, whether one accepts the invitation to a health prevention intervention now or refuses in the hope of receiving treatment later when acute health problems appear. In none of the cases is there “the right solution”, with each decision one does potentially something wrong. These are smaller and larger life dilemmas, which life presents to all people, on a daily basis [4, 7, 10]. Some people suffer from such life problems and dilemmas, others can cope with them productively [5, 6, 11, 17]. Successful life coping depends largely on the degree of individual wisdom [6, 10, 17]. Wisdom can thus be a resilience factor in coping with life’s stresses, of which any are always present.

Wisdom is discussed concerning its overlaps with other concepts: For example, wisdom has been included in modern concepts of leadership [18]: Hereby the idea is that one needs intelligence, wisdom and creativity for being an effective leader, whereby intelligence is seen as the basis for wisdom. Research found certain overlaps between intelligence and wisdom, but not too robust correlation of formal knowledge or fluid intelligence on the one hand, and (the more complex and differently conceptualized) wisdom on the other hand [19, 20].

Wisdom has also been discussed in comparison to similar concepts, such as religiosity [21] or acceptance and commitment (ACT [22]). However, the difference is that wisdom is a multidimensional concept containing a broader set of capacities. The application of wisdom means a situation-specific choice of wise coping capacities which is then recognized by others as “a wise action”. In order to apply wisdom practically, knowledge of basic wisdom attitudes is needed.

### Wisdom requires a multidimensional set of capacities

Since life stresses and dilemma target different life aspects and topics (family, money, profession, living, etc.) which all may require different coping strategies, wisdom is understood as a multidimensional mental capacity [8, 9]. Summarizing and integrating different wisdom components [10], twelve different capacities were found to constitute wisdom. They are also relevant for training- and public health purposes. In the sense of a multidimensional concept, wisdom includes a realistic view of the world (factual knowledge, contextualism, value relativism), other people (change of perspective, empathy), one’s own person (entitlement relativization, self-relativization, self-distance), one’s own experience (emotion acceptance, serenity) and the future (tolerance of uncertainty, sustainability perspective), as well as the ability to accept conditions and translate them into forward-looking behavior [10].

Wisdom can be contrasted with rigid, dogmatic, and inflexible thinking and is thus partially incompatible with persisting in embitterment and inflexibility [4]. Wisdom is associated with the ability to think dialectically, with social or practical intelligence and creativity, with humor and empathy or with autonomy and growth orientation. Wisdom is associated with lower personal suffering, and the personality dimension openness to new experiences. There is no correlation between wisdom on the one side and the desire to behave wisely or with formal education on the other side. Wisdom is the best predictor of life satisfaction in both men and women and can offset the influence of negative age influences on life satisfaction [11]. Wisdom has a greater influence on life satisfaction in older adulthood than health, socioeconomic status, financial situation, environment, or social engagement.

This means that wisdom affinity and competencies are more important for life satisfaction than objective life conditions. People with high wisdom are better able to distance themselves from stressful events and thus calm down, to use active coping strategies for cognitive reassessment (“reframing”) or for coping with the life problem, and to apply gained life experiences in new problem situations. In contrast to “non-wise” persons, “wise” persons have the insight that it is not the external situation, but their own reaction that influences their well-being. Similarly, “wise” persons are more concerned with the well-being of others rather than with their own [1, 4, 11, 12, 13]. In the modern psychosocially and cognitively demanding world, a lack of wisdom and stress coping capacities is often accompanied by inability to work and a risk of losing one’s job. Therefore, capacity trainings received special attention in public health such as prevention and rehabilitation settings [5, 10].

Wisdom intervention research shows that teaching wisdom capacities can lead to relevant improvements in life coping and activity in persons with chronic illness [5, 7, 14, 15]. Without basic attitudes for wise problem solving, no wise problem-solving behavior is possible. Therefore, basic wisdom attitudes can be introduced conceptually. Then training of their application may be useful: This can be done by exercises in which a person has to choose and apply a behavior to a fictive life problem situation, or the training group discusses which wise behavior fits the complex problem situation.

### Research questions

Against this background, the question arises to what extent people in the general population agree with basic wisdom ideas, respective wisdom attitudes. Wisdom attitudes are necessary prerequisites for enabling wise actions. This present national representative study aims to investigate the distribution of wisdom attitudes across age groups, sex and socio-economic status.

1. First, representative data on wisdom affinity (i.e. agreement with different wisdom attitudes) shall give an insight on how many people in the general population are at risk for life coping problems due to lack of wisdom affinity, and thus could benefit from possible training.

Additionally, potentially existing pattern of characteristics shall be explored:

(2) Are there systematic associations between certain socio-demographic and health-related characteristics and global wisdom affinity? Is there a pattern of characteristics in any of the groups with low, moderate, or high wisdom affinity?

## Methods

### Procedure

The study examined a large population-representative sample of 2509 people, aged 16–95 years. The study was conducted by a professional social research company (USUMA GmbH Berlin), which has many years of expertise in population-based representative surveys [16].

The sampling procedure was a three-stage process: Sampling areas were selected by random sampling. USUMA works with 250 sample areas throughout Germany, so that 10 interviews can be realized per area. In a second stage, random selection of households was done within these areas, based on on-site inspection. In a third stage, the interviewer must then identify all households and select an interview person per household, by means of a predetermined random procedure using the “Swedish key”.

The surveys were conducted by means of interviews and self-report questionnaires with the respondents on site. Socio-demographic data and presence of chronic physical or mental disorders were assessed by interview. Wisdom attitudes were assessed using the 12-WD Wisdom self-rating Scale [10]. Ethics approval was obtained from the Technische Universität Braunschweig (D-2021-03).

### 12-WD wisdom scale

In this population-representative study, the 12-WD Wisdom Scale was used for assessing global wisdom affinity [10]. The 12-WD scale is a self-report questionnaire that measures general wisdom-related attitudes, self-perceptions and self-attributions. Participants were asked how strongly they agreed with 12 given wisdom statements, in a life problem situation. The instruction of the 12-WD scale is: “Below you will find very different statements and principles on how people can react to enormous difficulties and considerable life stresses. Please think of the situation just presented. For each statement, decide to what extent it makes sense or not to you personally in this situation.” Responses are given per item on a Likert scale from 0 (strongly disagree) to 10 (strongly agree). Each of the twelve statements represents one of the twelve theoretically based wisdom capacities, and they can be grouped into five larger domains:

#### View on the world

(1) Factual and procedural knowledge: General and specific knowledge about problems, what constitutes problems, and the possibilities of solving them; (2) Contextualism: knowledge about the temporal and situational character of problems and the numerous conditions in which life is embedded; (3) Value relativism: knowledge of the diversity of values and life goals and the need to look at other people within their value system without losing sight of one’s own values.

#### View of other people

(4) Change of perspective: the ability to describe a problem from the perspective of other people. (5) Empathy: the ability to understand and feel the emotional experience of another person.

#### View of one’s own person

(6) Relativization of problems and aspirations: The ability to be humble and accept that one’s problems may not be that important compared to many problems in the world. (7) Self-relativization: the ability to accept that one is not always the most important individual and that most things do not follow one’s will or are not aligned with one’s interests. (8) Self-distancing: The ability to recognize and understand the perception and evaluation of oneself from the perspective of other people.

#### View of one’s own emotional experience

(9) Perception and acceptance of emotions: The ability to recognize and accept one’s own emotions. (10) Emotional composure and humor: the ability to be emotionally balanced, to control one’s own emotions depending on the situation, and the ability to view oneself and one’s own difficulties with humor.

#### View to the future

(11) Sustainability: The knowledge of short- and long-term consequences, which can contradict each other. (12) Tolerance of uncertainty: knowledge and acceptance of the fact that future developments can never be reliably predicted or controlled.

A global wisdom score can be calculated as a mean value across all 12 items of the 12-WD Wisdom Scale. The idea is that a person has a conceptual understanding of these different wisdom ideas, and only then can be ready to act wisely, according to some of these world views, when it comes to difficult or ambiguous life situations. Cronbach´s alpha for the 12-WD Scale was 0.868 in the present investigation. In order to achieve sufficient differentiation in the items´ rating, and because positively formulated attitude items tend to be answered positively, we adjusted the rating scale from the original scale of 1 to 6 [10] to a scale of 0 to 10 for each item.

For interpretation of a person´s global wisdom score, a broad categorization into low, moderate, and high wisdom affinity is useful. For the present analysis, this categorization into low, moderate, high wisdom affinity is done according to the following conceptual and empirical aspects:

“Low wisdom affinity” was originally defined by the authors of the Wisdom Scale [10] as below-average mean values < 3.5 (on a scale 1–6). Transferred to the scale used in the present investigation, “low wisdom affinity” is thus assumed for 12-WD mean scores < 5 on a scale of 0–10.

**Fig. 1:**
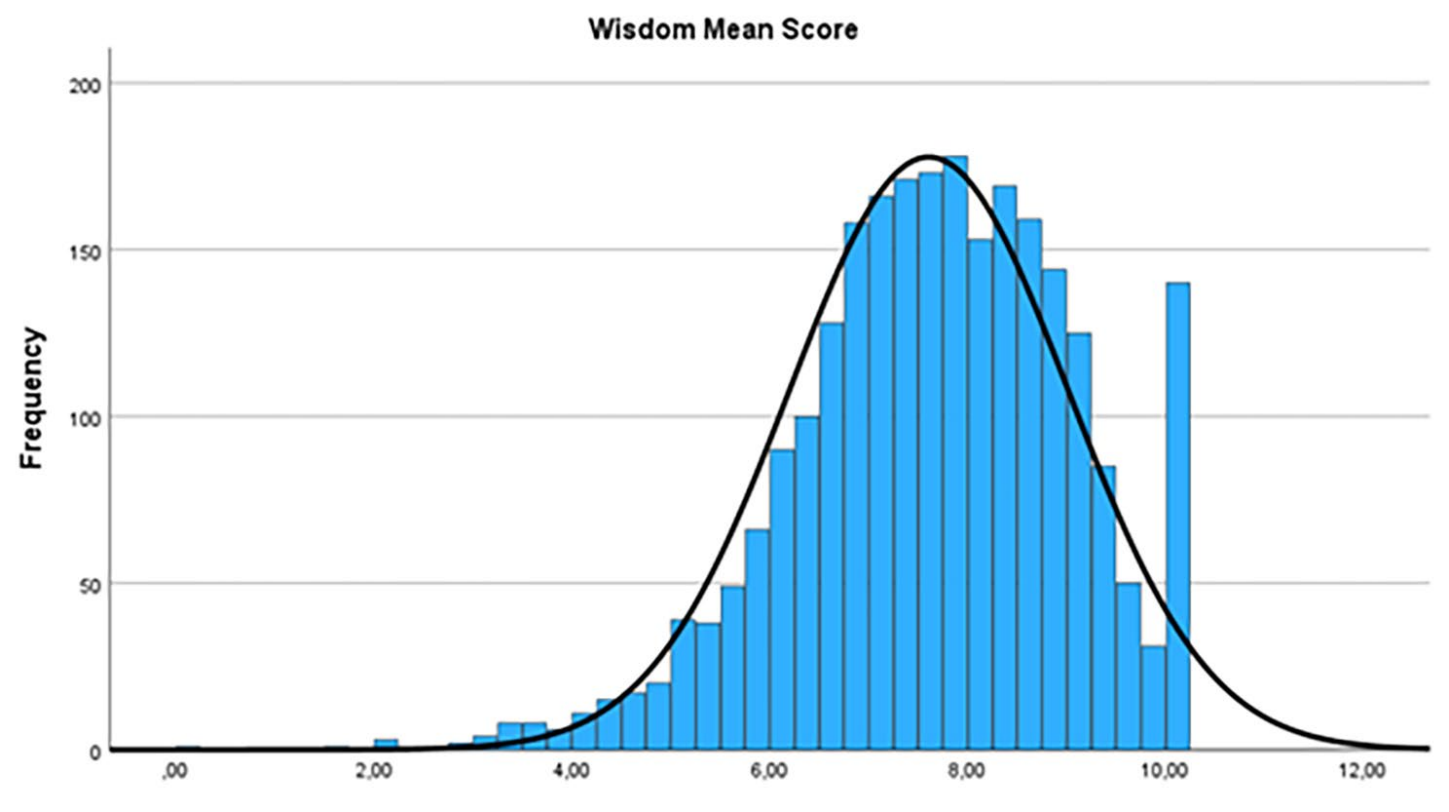
Distribution of global wisdom mean scores according to the 12-WD Wisdom Scale [10] from the representative sample (*N* = 2509)

“High wisdom affinity” is defined according to the distribution of raw values of the Wisdom Scale in this present investigation: A relevant number of people reported highest possible agreement on the different wisdom items, i.e. ratings of “10” (on the scale 0–10). With regard to the empirical distribution of the ratings (Fig. 1), and the literature-based basic assumption that there are few people with high (5% [17]) and few people with low agreement to the wisdom ideas (7%, [10]), only people with highest possible agreement (i.e. rating “10”) were grouped as those with high wisdom affinity.

In sum, this results in interpretation of the 12-WD mean values in the present study as “low (rating 0–4)”, or “moderate (rating 5–9)”, or “high (rating 10)” wisdom affinity.

### Chronic mental and physical illness

Mental illness was assessed by concretely asking for mental health problems with accompanying impairments: “Do you regularly have - now and earlier - complaints such as anxiety, mood or interactional problems, which lead to impairments in your daily life routines? Have you been in treatment because of these problems, or have others suggested that you should go to treatment (at physicians, or psychologists)?” We have already asked about mental illness globally in this way in various other studies [23, 24]. The items are content-valid, comprehensible and have been validated in comparison to a standardized interview [23].

The survey contained basic socio-demographic data, as well as questions about mental illness with regular impairments and need for treatment. Since mental illnesses are usually chronic and recur over the life span, with relapses or even continuous problems [23, 25–27], a distinction between acute and chronic becomes obsolete here. What is of interest is whether someone is repeatedly confronted with treatment-prone mental health problems that impair them in their daily life.

### Statistical analysis

Descriptives are calculated (mean values, standard deviations, frequencies), as well as group comparisons (according to low/moderate/ high wisdom affinity) by *X*^2^ or T-test. An exploratory regression analysis was conducted, for investigating the correlation pattern of socio-demographics with global wisdom affinity.

### Participants

2509 people from the general population in Germany were surveyed in 2021 (Rep33, USUMA, 2021). Data collection was carried out by USUMA GmbH. The sample contained 49% men and 50.9% women, and three persons (0.1%) who identified themselves as divers. A higher school education (12 years, i.e. Abitur/A-Levels) were reported by 22.5%, 95.2% were presently in any employment. 59.8% of the participants had a partnership, 34.3% lived in a single household. 69.6% had any religious denomination. 4.9% had no own income, 44.1% had an own monthly income up to 1500 €, and 51% more than 1500 €.

## Results

Overall, 90% of all investigated supported the wisdom ideas positively (with 12-WD scale means between 5 and 9 on a scale from 0 to 10). Only 6% of the whole sample appeared to be highly wisdom-affirmative (12-WD mean score 10), whereas 4% may appear “unwise” due to very low agreement (12 WD mean score 0–4).

The exploratory regression analysis (Table 1) indicated that sex, age, socioeconomic and living situation (income, unemployment, town size), religious denomination, and chronic illness all had certain explanatory value for wisdom. However, correlations were rather low, and statistical significances can easily appear due to large sample size.

Higher school education and household situation were not associated with the global wisdom affinity.

**Table 1:** Correlates of wisdom affinity in a national representative sample (*N* = 2431). Stepwise linear regression analysis, inclusion method, 4th step corrected R^2^ = 0.055, significant change in F *p* < .001

Characteristics	Wisdom attitudes mean score (12-WD-Scale)	
	Beta coefficient	Significance level *p*
Sex (male = 0, divers = 1, female = 2)	0.097	< 0.001
Age in years	0.056	0.012
School degree A-levels (no = 0 yes = 1)	− 0.017	0.422
How often unemployed during one´s life	− 0.085	< 0.001
Own monthly income in €	0.093	< 0.001
Number of persons living in the household	0.025	0.250
Town size (number of inhabitants)	− 0.050	0.012
Religious denomination (no = 0 yes = 1)	0.040	0.044
Chronic mental disorder (no = 0 yes = 1)	− 0.098	< 0.001
Chronic physical illness (no = 0 yes = 1)	− 0.096	< 0.001

For better more detailed understanding of people with low, moderate, or high wisdom affinity, the three groups were compared (Table 2). The question was whether there are differently distributed characteristics in people with different degrees of wisdom affinity. In this explorative analysis, some interesting patterns came up:

First, distribution of gender and age, as well as household situations were not significantly different in the three groups.

Some differences were seen in socioeconomic, religious and health aspects:

The highly wisdom affirmative group reported more often middle to higher monthly incomes (1000–3500 €) as compared to those with lowest wisdom affinity. The highly wisdom affirmative had less often been unemployed during their life. Most of the highly wisdom affirmative lived in towns of middle sizes, whereas the low and moderately wise lived rather in smaller (< 100.000) or very large cities with > 500.000 inhabitants.

Most of the moderately wisdom affirmative had a religious denomination (70.9%), whereas only 57–59% of the highest or low wisdom affinity reported religious affiliations.

Low wisdom affirmative were most often chronically ill (25%), with mental or physical illness in similar frequency. Only 11–14% of the moderately wisdom affirmative, and 3–5% of the highly wisdom affirmative reported chronic illnesses.

**Table 2:** Comparison of persons with high, moderate and low wisdom affinity according to 12-WD wisdom scale mean score. Percentages or means (standard deviation) are reported (ANOVA or *X*^2^-Test, *N* = 2509)

Characteristics	Low Wisdom Affinity L (*n* = 97)	Moderate Wisdom Affinity M (*n* = 2272)	High Wisdom Affinity H (*n* = 140)	Significance level *p* in group comparison (ANOVA overall and Post-Hoc tests, or X^2^)	All (*N* = 2509)
Sex (male = 0, divers = 1, female = 2)	M: 56.7% D: 0.0% F: 43.3%	M: 48.6% D: 0.1% F: 51.3%	M: 50.7% D: 0.7% F: 48.6%	0.130	M: 49.0% D: 0.1% F: 50.9%
Age in years	47.54 (19.7)	49.39 (17.8)	52.22 (17.0)	0.104 (ANOVA)	49.48 (17.8)
School degree A-levels (no = 0 yes = 1)	14.4%	23.1%	19.3%	0.088	22.5%
How often unemployed during one´s life At least once unemployed	1.54 (2.17) 52.1%	0.99 (1.61) 45.1%	0.59 (0.88) 37.1%	< 0.001 (ANOVA) L vs. M 0.003 L vs. H < 0.001 M vs. H 0.011	0.99 (1.60) 44.9%
Own monthly income in €				< 0.001	
No own income < 500 € 500-1.000 1.000–2.000 2.000-3.500 > 3.500	8.5% 4.3% 25.5% 37.3% 22.4% 2.1%	4.9% 3.3% 14.5% 46.7% 26.6% 4.2%	2.9% 0.0% 12.2% 55.8% 25.2% 4.3%		4.9% 2.9% 14.9% 46.8% 26.4% 4.1%
Number of persons living in the household	1.97 (1.25)	2.13 (1.12)	2.06 (1.03)	0.304 (ANOVA)	2.08 (1.02)
Town size (number of inhabitants)				< 0.001	
< 2.000 2.000–20.000 20.000-100.000 100.000-500.000 > 500.000	11.3% 24.8% 23.7% 15.5% 24.7%	9.1% 33.0% 24.8% 14.7% 18.5%	13.6% 27.1% 12.9% 40.7% 5.7%		9.4% 32.3% 24.1% 16.2% 18.0%
Religious denomination (no = 0 yes = 1)	59.4%	70.9%	56.8%	< 0.001	69.6%
Chronic mental illness (no = 0 yes = 1)	24.7%	10.9%	2.9%	< 0.001	11.0%
Chronic physical illness (no = 0 yes = 1)	25.8%	13.8%	5.1%	< 0.001	13.8%

## Discussion

The representative survey brought interesting findings according to our research questions:

First, a relevant number of about 4% of respondents, who had lowest wisdom affinity, may constitute a group in need of wisdom and coping training, especially as these are in one fourth of cases impaired by chronic illnesses, i.e. enduring real-life problems. The frequency of persons with low wisdom affinity is similar with the rate of people with severe work anxiety, embitterment reactions, or broad psychological capacity deficits in previous representative surveys [28].

Second, it is obvious that the group with low wisdom affinity appears with several accompanying problems: Chronic physical and mental illness are more often present in these people with lower wisdom affinity, and work problems (here especially proportion of unemployed) also appear associated with lower wisdom affinity. The rate of impairing mental disorders in the low wisdom affirmative group is relevantly higher (25%) than in the two more wisdom affirmative groups (3–11%). In this present investigation, “mental illness in treatment or prone for treatment” has been assessed. This leads to a conservative estimation of mental illness, as it may ignore subsyndromal conditions. Epidemiological research based on standardized interviews consistently over decades found rates about 30% when asking for mental illnesses only (independent from treatment) [29]. These data, however, must be expected to contain some false positives due to methodological artefacts [30].

There are good reasons why people with mental illness are more prone for wisdom problems than others: People with mental illnesses regularly have problems in coping with special life problems [31]. Negative situational and self-perceptions, depressive mood and worrying are typical psychopathologies in mental disorders which can impair clear reasoning and situational-oriented coping [5, 16, 31–34]. Also, rambling, circumstantial thinking which can be found in patients with brain-organic and (schizo)affective disorders, can then lead to difficulty in decision making, e.g. distinguishing for important or unimportant content [33]. These problems can be hindrances in many everyday problems and complex situations, e.g. dealing with finances, purchases, prioritizing and completing tasks, and especially work ability [31].

An associated finding is that the low wisdom affirmative persons have more often been unemployed (> 50% of cases at least once) than the other groups. Employment is an important basic need, in order to be socially involved, have a certain task, and get feedback and reputation. People suffer from unemployment due to the loss of social net, and additional financial restrictions [35]. The belief in one’s own capacities can be lost, and wisdom capacities can be blocked. In cases of injustice along with stressful job events, e.g. after being fired from one´s job unjustly, an embitterment reaction can emerge. Embitterment is often coming along with blocked wisdom capacities. In such cases wisdom training has been found to be helpful [5], in order to reactivate these capacities. Beside higher unemployment rates, the socio-economic condition of persons with lower wisdom affinity was weaker. This fits to the known connection between mental illness and work ability problems, which often come along with lower socioeconomic status.

Wisdom seems to be rather independent from the age and gender: The finding that age and gender was similarly distributed in the groups with low, moderate, and high wisdom affinity support the results from a previous study [10]. There, only a small positive correlation was found for wisdom affinity and age (*r* = .15, *p* = .04). Age and wisdom have been discussed quite often until now [6, 36, 37], with different findings on whether wisdom is growing with ageing. Most studies speak for a curvilinear association with growing wisdom in middle age, and potentially decline in very old age in case cognitive deficits emerge [6]. Throughout the lifespan, there are significant events, upheavals, innovations, adjustment needs, and problems which all require different and specific coping capacities. Consequently, a wide range of wisdom capacities are required and can be deployed and learned at different age [38]. Some naturally emerging increase in wisdom over the life span [39] seems normal, due to collecting experiences with different life problems. There were no gender differences in the global wisdom affinity in our representative sample. Similarly, previous research showed hardly gender differences in wisdom [6, 10, 40, 41]. Thus, a similar global wisdom affinity should be assumed for men and women.

An interesting pattern in the moderate wisdom degree group is that 71% had any religious affiliation – significantly more than in both the highest and lowest wisdom affinity groups (57–59%). Thus, religiously affiliated persons are overrepresented in the group who reflected the wisdom items with some differentiation, i.e. who did agree, but not fully agree to all items (as did the group scoring “10”). Religion and wisdom have some conceptual overlap: they contain by their nature relational and dialectic ideas, e.g. that what is right or wrong depends on each concrete situation, requiring situation analysis, moral and ethical decisions, and sometimes trust into somebody or something. Qualitative content validation of the wisdom scale recently showed that people give very differentiated ideas when they are asked concerning the meanings of the 12 wisdom items [15]. Thus, a person who agreed to most of the 12 wisdom ideas, but not fully (rating < 10), might have reasons for his moderate agreement, e.g. “empathy is a useful mean, but in non-interactional situations factual knowledge may be more needed.” Religion contains relational dialectic understanding of the world. Thus, it may be reasonable that especially people with religious affiliation give moderate (instead of full agreement) ratings on the wisdom scale. Associations between spirituality or religion and wisdom have been discussed in the scientific literature [21], and religiosity was seen as an alternative pathway to well-being, with partly overlap of wisdom ideas, religiosity, and mastery or purpose in life [21].

### Limitations and methodological aspects

The representative data are based on self-assessments of the interviewed by means of questionnaires which is only one aspect of wisdom assessment [42]. Therefore, no conclusion can be drawn concerning wisdom-oriented coping in real life situations. This would require situational observations. These, however, are not suitable in the context of a population-based representative survey with large age range. Also, they could only test a few selected standardized problem situations [43].

Inducing a focus on one´s own past behavior by the questionnaire´s instruction, an attempt was made to counteract the effect of the global approval tendency, and to obtain more differentiated answers. The broad scaling (visual analogue scale with 11 scale points) was chosen to enable better differentiation. The different distributions of the wisdom affinity scores (encompassing the scale range fully from 0 to 10) indicate that this differentiation was senseful. The distribution of wisdom affinity scores is a right-steep normal distribution (in contrast to a two-peaks distribution which is often seen when distributions of clinical symptoms are shown) which is an additional advice for concept validity of wisdom as a capacity and not a clinical symptom entity.

The effects of healthy self-overestimation and the intention-behavior gap [44] may have influenced the results. It is possible that the interviewed overestimated their own possibilities for action according to the wisdom contents, or they might have expressed their desire for such wisdom performance (rather than their realistic earlier actions). It can be assumed, that in reality, when dealing with concrete life problems, not all people would act according to the principles they agreed to.

The data thus indicate that people tend to agree with the wisdom principles to varying, mostly moderate degrees, and think that they themselves act accordingly. Thus, the groups of low agreement, moderate agreement and full agreement can be interpreted as people with a more or less pronounced affinity for basic wisdom ideas.

## Conclusion and outlook

The representative data for the first time show a distribution of self-reported wisdom affinity in a general population. Some people were found to have a low affinity for wisdom, including people with socio-medical risk factors such as unemployment and chronic mental health problems. It must be assumed that people with socio-medical risk factors also have impairments in their wisdom-related problem-solving strategies, and that these can be of interest for transdiagnostic wisdom trainings in prevention [15, 45] or rehabilitation [5], which has already shown positive effects. The wisdom ideas of the 12-WD Wisdom Scale can be used in trainings in clinical and non-clinical settings [5, 15].

## Data Availability

These can be made available from the author of this research article, Beate Muschalla, upon request.
